# Supplementary material for: Systematic Review and Meta-Analysis of Oral Anticoagulant Therapy in Atrial Fibrillation Cancer Patients
Source: Cancers (Basel). 2023 Apr 30;15(9):2574. doi: 10.3390/cancers15092574 (PMC10177228; doi:10.3390/cancers15092574)
Supplement: Supplementary file 1 [file cancers-15-02574-s001.zip › cancers-2336730-supplementary.pdf]

Supplementary Material

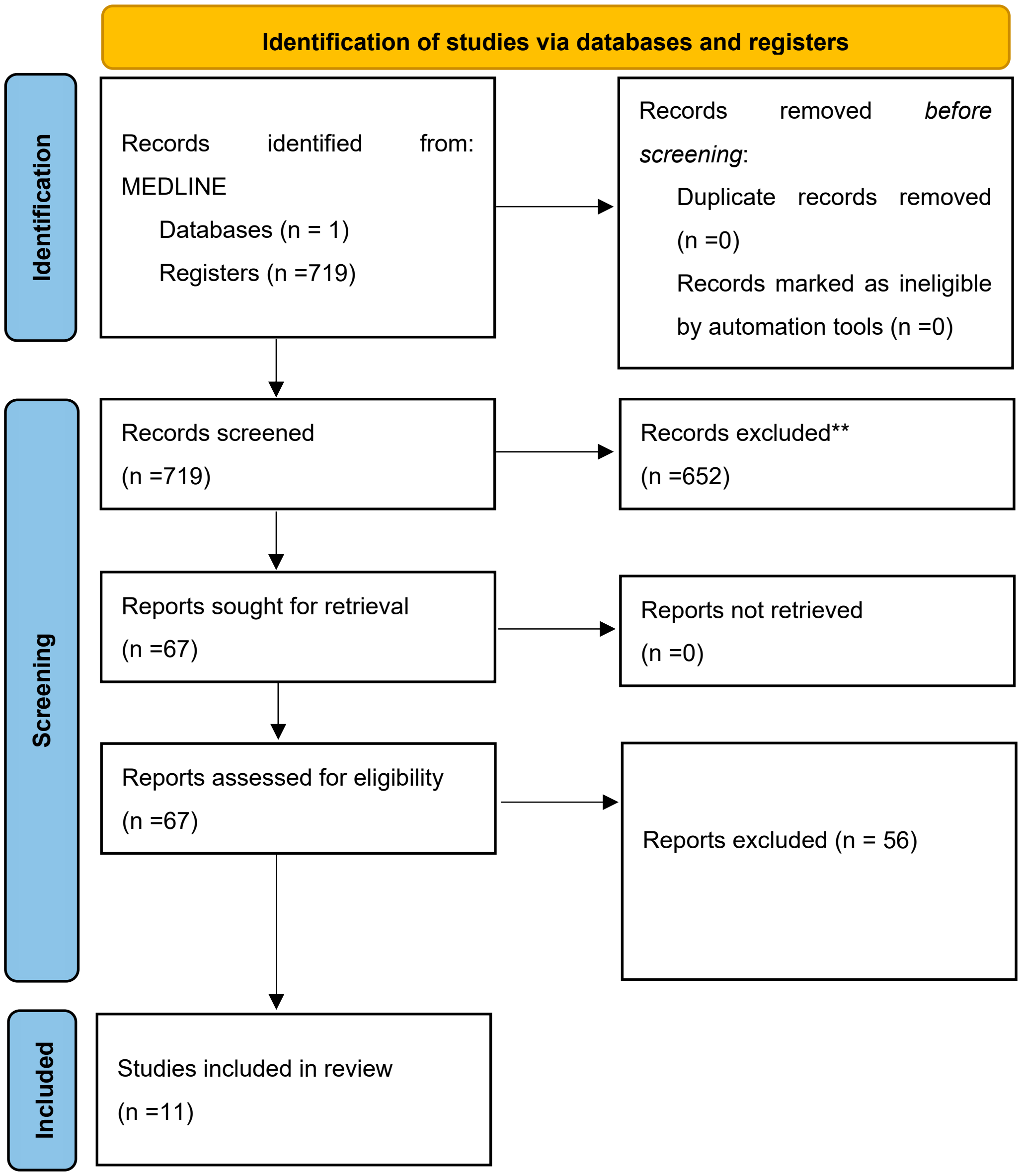

Figure S1. PRISMA 2020 flow diagram for new systematic reviews which included searches of databases and registers only.

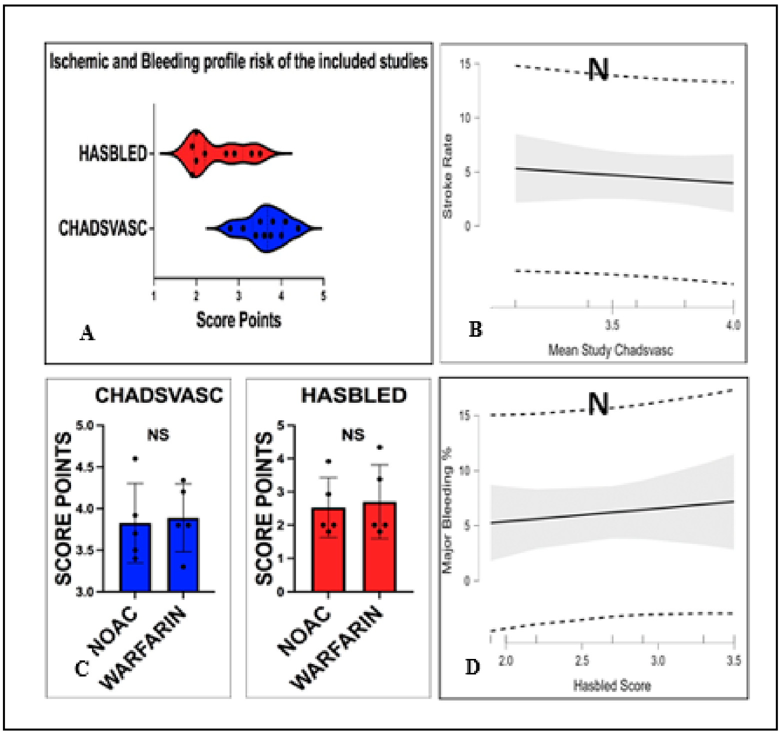

Figure S2. A. Mean value of the Has-Bled and Chadsvasc score points in the included studies. B. Nonsignificant relationship between stroke and Chadsvasc. C. Has-Bled and Chadsvasc difference between patients on NOAC and Warfarin therapy. D. Nonsignificant relationship between Major Bleeding and Has-Bled.

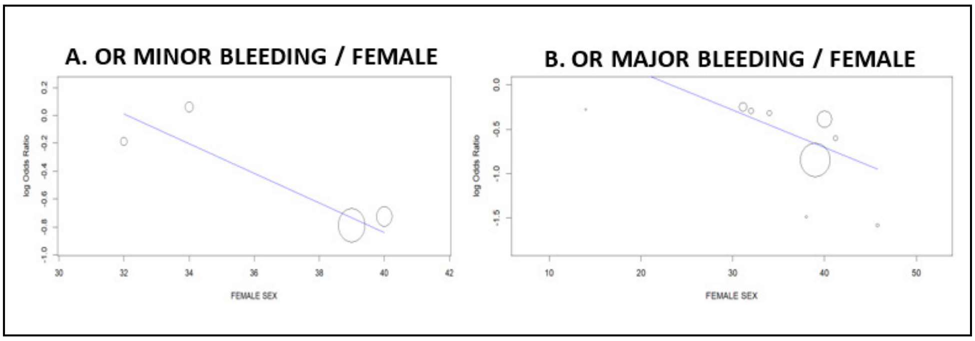

Figure S3. A. Meta-regression OR Minor Bleeding / Female Sex. B. Meta-regression OR Major Bleeding / Female Sex.

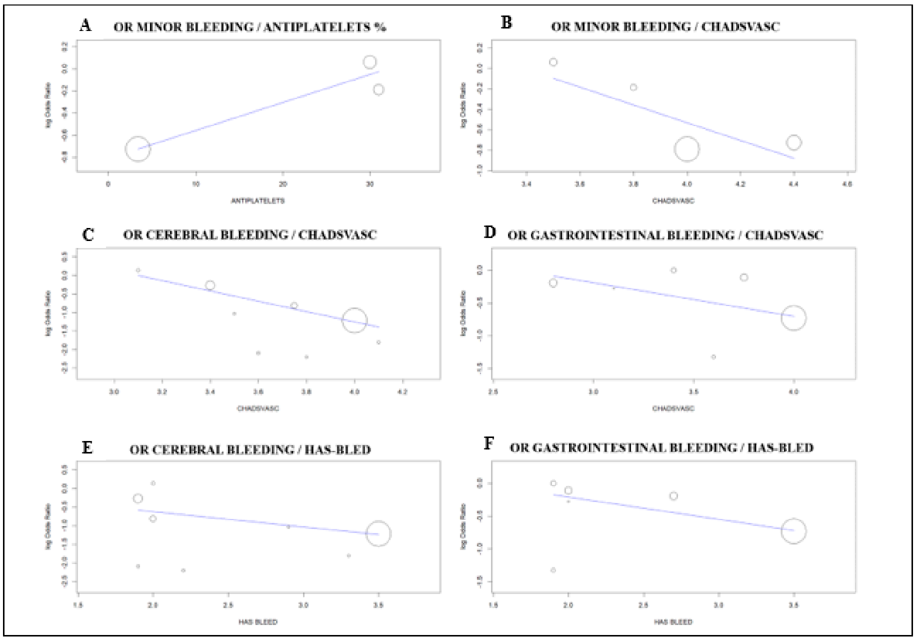

Figure S4. A. Meta-regression OR Minor Bleeding / Antiplatelets. B. Meta-regression OR Minor Bleeding / Chadsvasc. C. Meta-regression OR Cerebral Bleeding / Chadsvasc. D. Meta-regression OR GI Bleeding / Chadsvasc. E. Meta-regression OR Cerebral Bleeding / Has.Bled. F. Meta-regression OR GI Bleeding / Has-Bled.

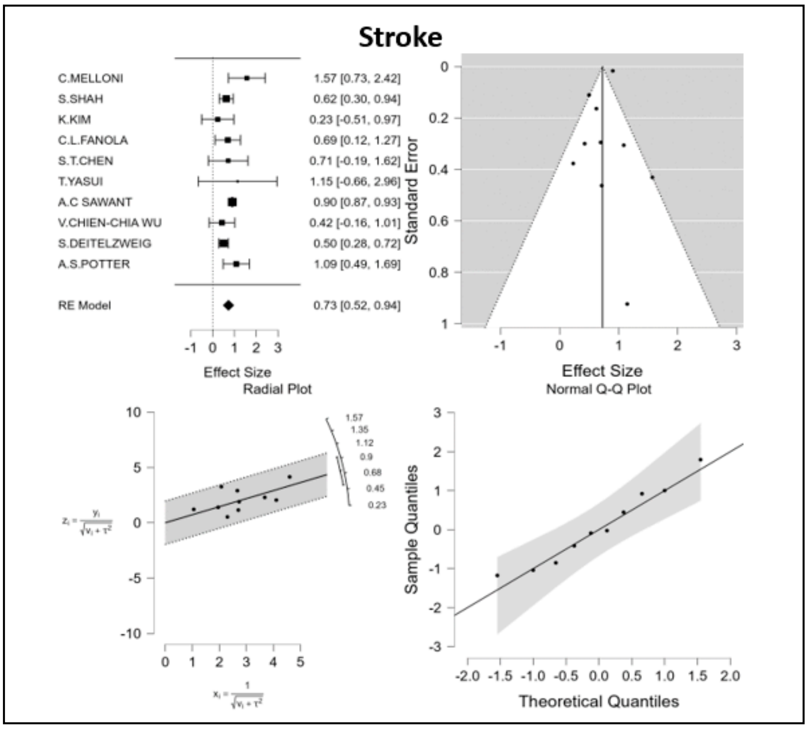

Figure S5. Model fit and Funnel Plot of stroke meta-analysis.

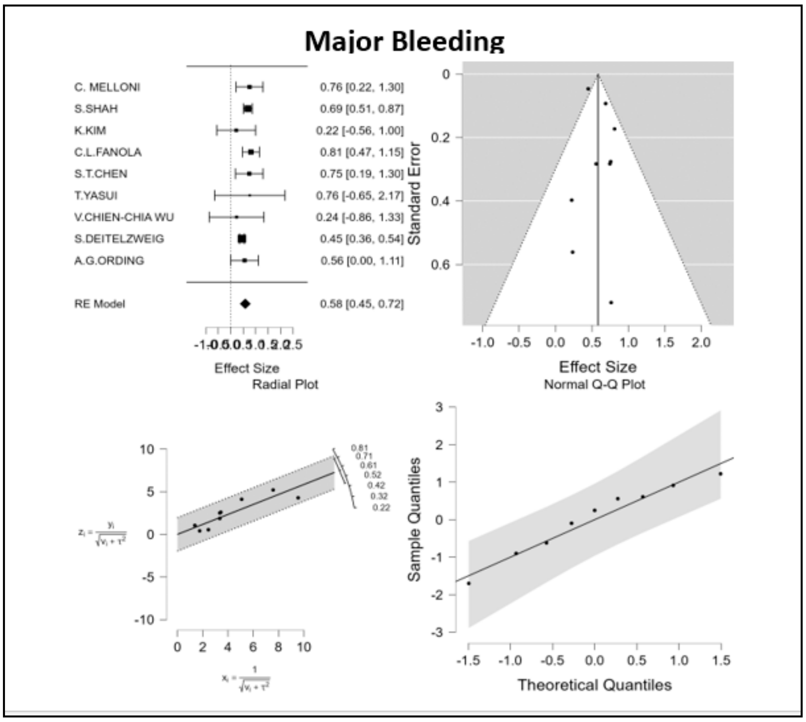

Figure S6. Model fit and Funnel Plot of Major Bleeding meta-analysis.

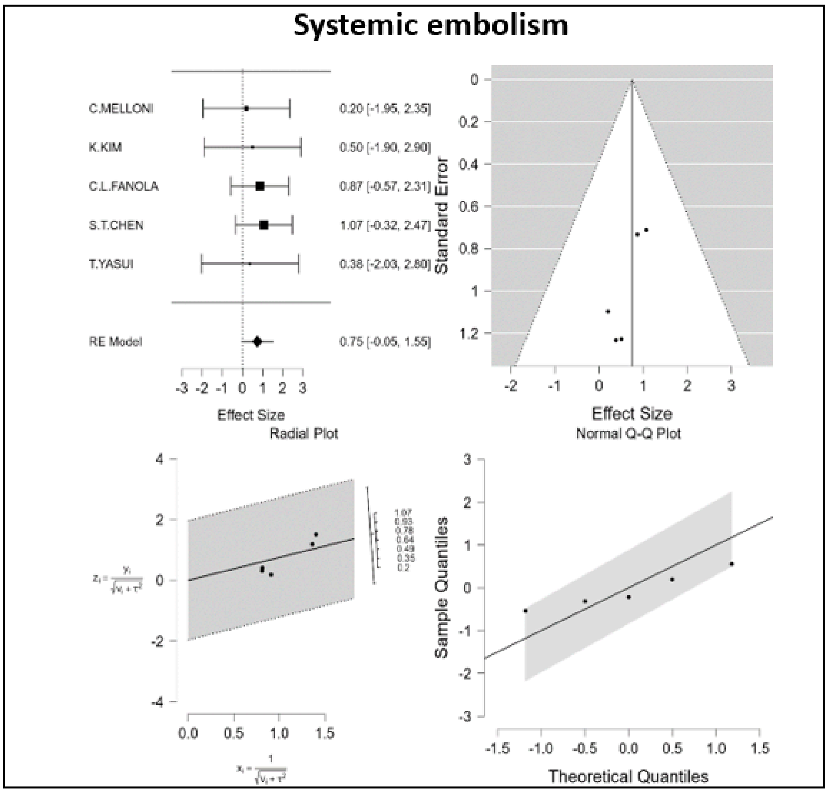

Figure S7. Model fit and Funnel Plot of Systemic embolism meta-analysis.

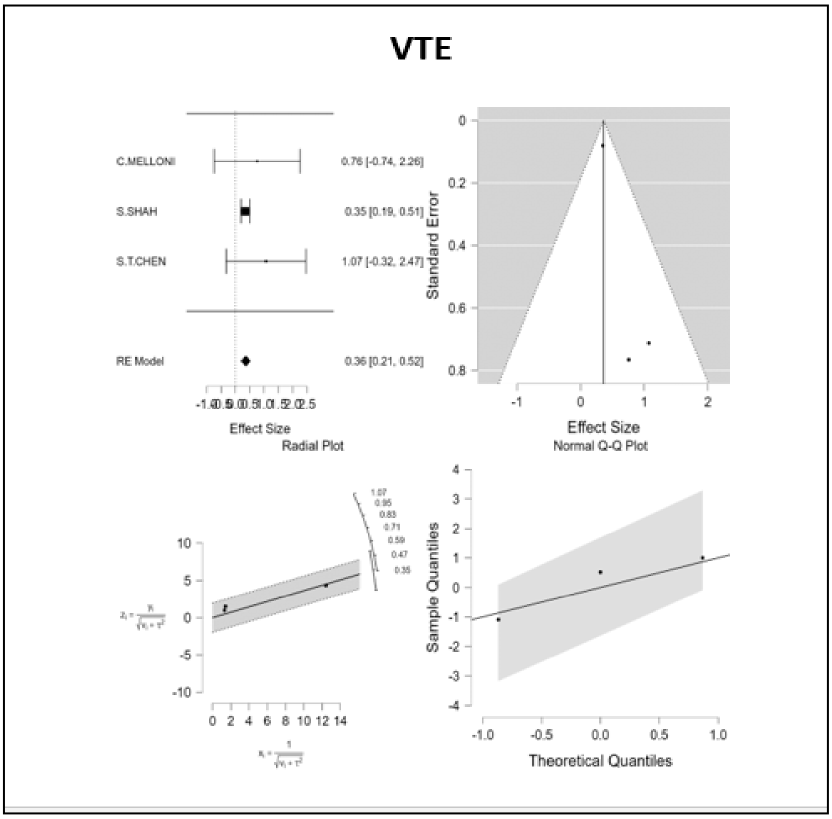

Figure S8. Model fit and Funnel Plot of VTE meta-analysis.

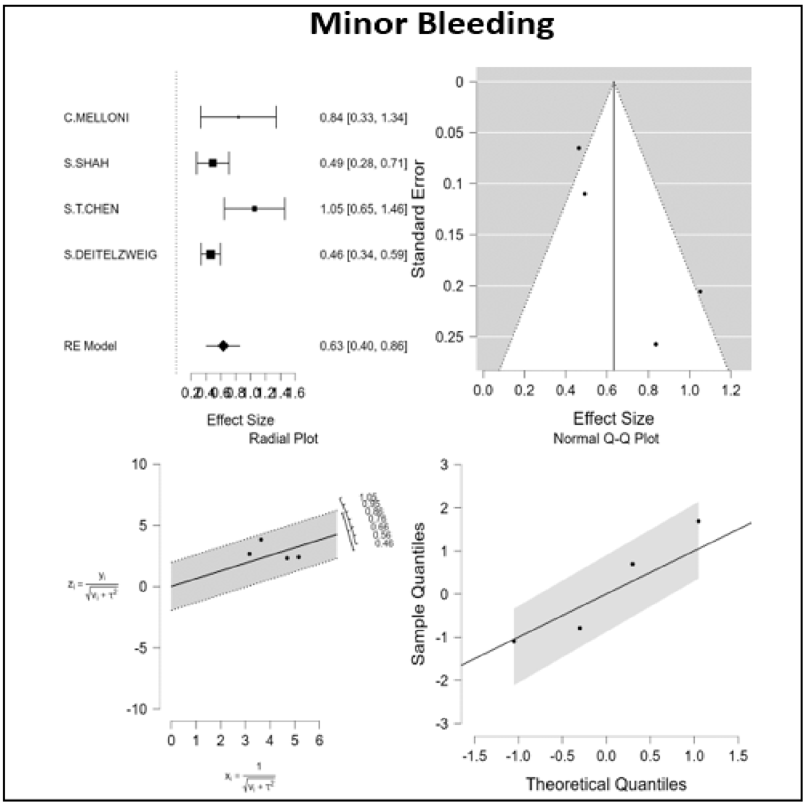

Figure S9. Model fit and Funnel Plot of Minor Bleeding meta-analysis.

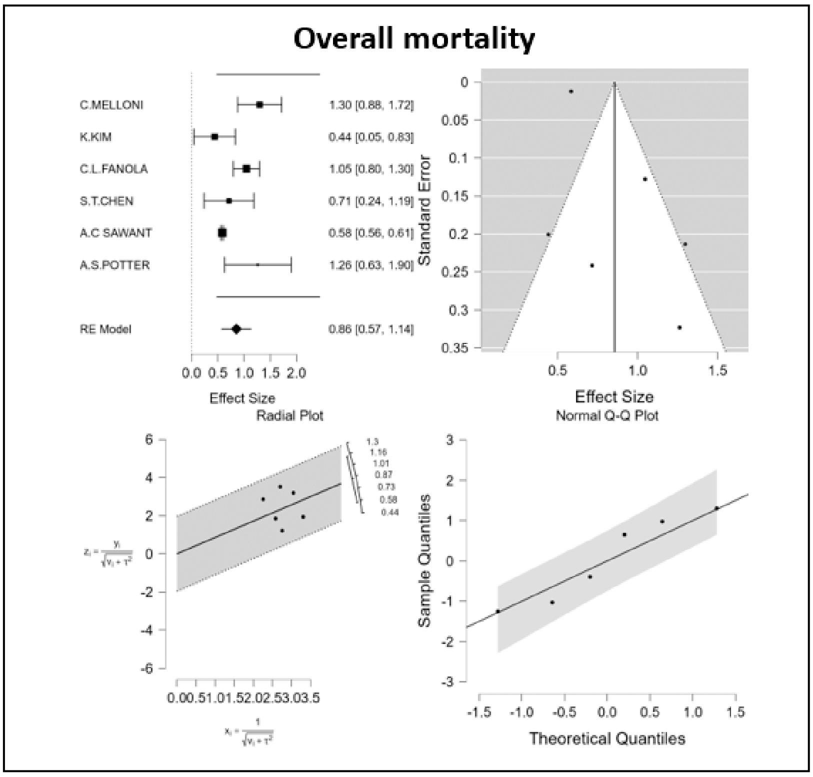

Figure S10. Model fit and Funnel Plot of overall mortality meta-analysis.

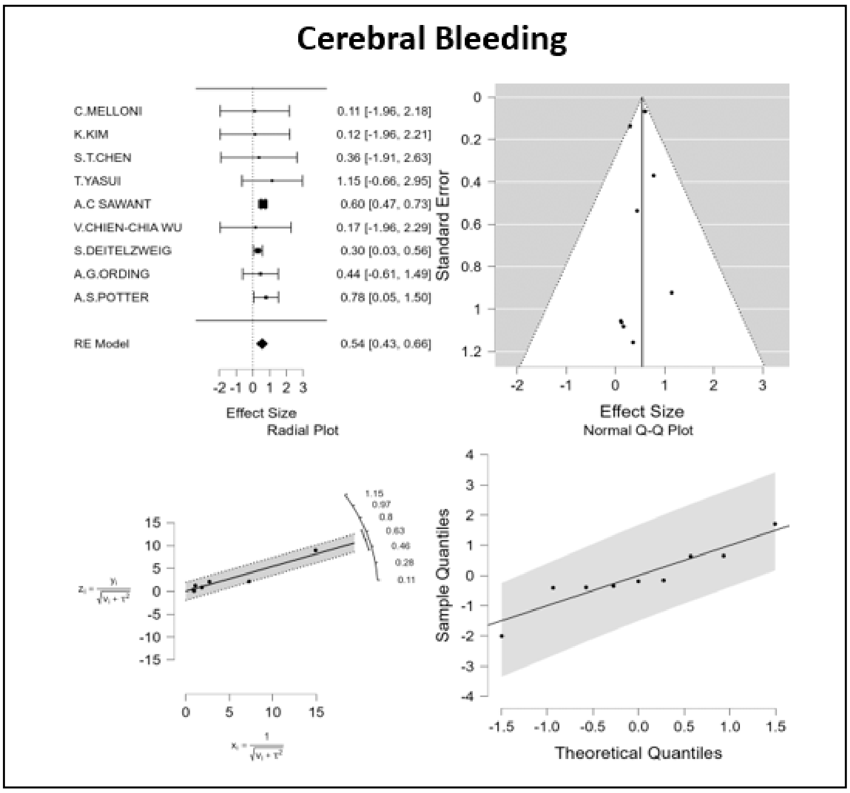

Figure S11. Model fit and Funnel Plot of Cerebral Bleeding meta-analysis.

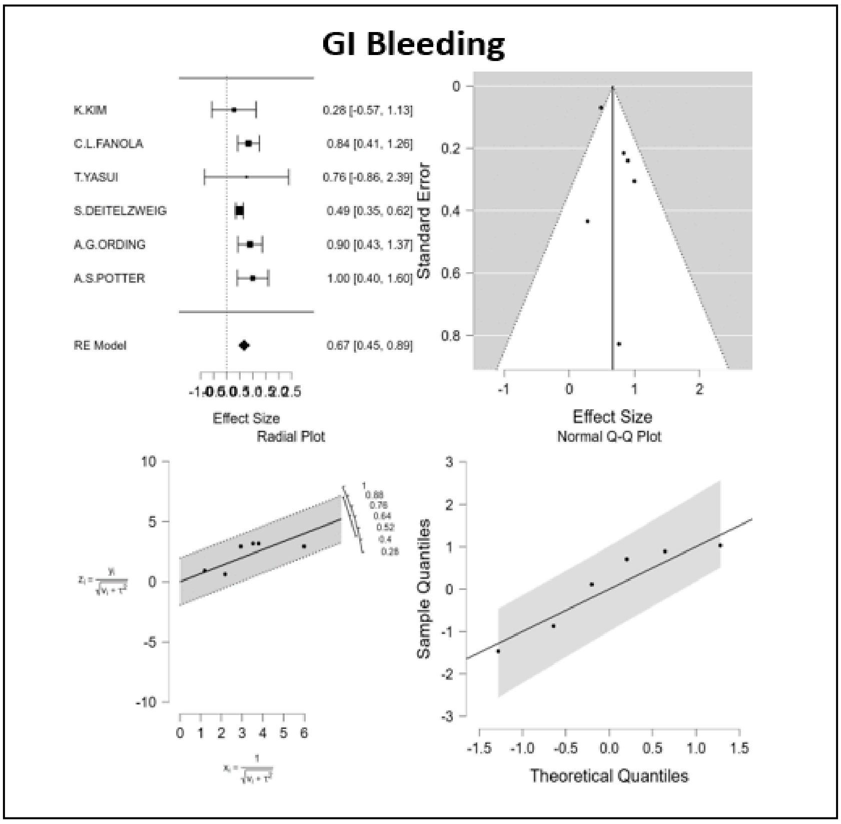

Figure S12. Model fit and Funnel Plot of GI Bleeding meta-analysis.

Table S1. Summary table of the studies included in the meta-analysis.

Authors, Journal of publication	Title	Sample size (N)	Study design	Variable-outcome	Main findings	Quality of evidence
Melloni et al. *The American Journal of Medicine (2017)*	A Efficacy and Safety of Apixaban Versus Warfarin in Patients with Atrial Fibrillation and a History of Cancer: Insights from the ARISTOTLE Trial	1236	Prospective, subanalysis from the ARISTOTLE Trial	Associations between history of cancer and stroke/systemic embolism, major bleeding, or death<br><br>CACS-composite endpoint (of stroke/systemic embolism, myocardial infarction, and death) apixaban vs warfarin	Cancer was not associated with a higher risk of stroke/systemic embolism, major bleeding or death<br><br>Superior efficacy and safety of apixaban versus warfarin were consistent in patients with and without cancer.	Medium
Sean T. Chen et al. *European Heart Journal* (2018)	Efficacy and safety of rivaroxaban vs. warfarin in	640	Prospective, subanalysis from the ROCKET AF	Associations between cancer history and stroke, venous	History of cancer was associated with higher rates of overall bleeding and non-cardiovascular death	Medium

Authors, Journal of publication	Title	Sample size (N)	Study design	Variable-outcome	Main findings	Quality of evidence
	patients with non-valvular atrial fibrillation and a history of cancer: observations from ROCKET AF			thromboembolism, or myocardial infarction and overall bleeding.<br><br>CACS-composite endpoint (of stroke/systemic embolism, myocardial infarction, and death) rivaroxaban vs warfarin	Relative efficacy of rivaroxaban vs. warfarin was similar in those with and without a history of cancer.	
Fanola et al. *Journal of the American Heart Association* (2018)	Efficacy and Safety of Edoxaban in Patients With Active Malignancy and Atrial Fibrillation: Analysis of the ENGAGE AF-TIMI 48 Trial	1153	Prospective, subanalysis from the ENGAGE AF-TIMI 48 Trial	Associations between cancer history and stroke, venous thromboembolism, or myocardial infarction and major bleeding.<br><br>Safety and efficacy profiles of edoxaban vs warfarin in active cancer	Malignancy was associated with an increased risk of all-cause death and major bleeding, but not stroke or SEE.<br><br>The efficacy and safety profiles of edoxaban compared with warfarin were similar in those who developed active malignancy compared with those who did not.	Medium
Kim et al. *Korean Circulation Journal* (2018)	Effect of Non-vitamin K Antagonist Oral Anticoagulants in Atrial Fibrillation Patients with Newly Diagnosed Cancer	1651	Retrospective, non-randomized.	Ischemic stroke/systemic embolism (SE), major bleeding, and all-cause death. Based on propensity score matching, 388 matched pairs were included in the NOAC and warfarin groups.	NOAC group had significantly lower incidences of ischemic stroke/SE ($p<0.001$), major bleeding ($p<0.001$), and all-cause death ($p<0.001$) than the warfarin group. Incidence of major bleeding was significantly lower in the NOAC group than in the warfarin group.	Medium

Authors, Journal of publication	Title	Sample size (N)	Study design	Variable-outcome	Main findings	Quality of evidence
Shah et al. *American Society of Hematology* (2018)	Comparative effectiveness of direct oral anticoagulants and warfarin in patients with cancer and atrial fibrillation.	16096	Retrospective, claims datebase based.	Primary outcome included ischemic stroke, severe bleeding, other bleeding, and venous thromboembolism (VTE) betwenn NOAC and Warfarin.	DOAC users experienced lower or similar rates of bleeding and stroke compared with warfarin users, and a lower rate of incident VTE.	Medium
Sawant et al. *Journal of Geriatric Cardiology* (2019)	Superior safety of direct oral anticoagulants compared to Warfarin in patients with atrial fibrillation and underlying cancer: a national veterans affairs database study	196521	Retrospective observational study performed utilizing the national VA Healthcare data.	Primary outcome included mortality, ischemic stroke, severe bleeding,, and venous thromboembolism (VTE) betwenn NOAC and Warfarin.	Superior safety profile of DOACs compared to warfarin among patients with underlying cancer and AF. Warfarin was associated with higher mortality, similar ischemic stroke risk but higher risk of hemorrhagic stroke.	Medium
Yasui et al. *Internal Medcine* (2019)	Oral Anticoagulants in Japanese Patients with Atrial Fibrillation and Active Cancer.	224	Retrospective Cohort Study	Rates of mortality, stroke, systemic embolism major bleeding between among Japanese cancer patients with AF receiving OACs.	Stroke or systemic embolism rate was a 2.8%/year in DOACS group while rate in the warfarin group was 5.4%/year. Major bleeding in the DOAC group was	Medium

Authors, Journal of publication	Title	Sample size (N)	Study design	Variable-outcome	Main findings	Quality of evidence
					4.0%/year and in the warfarin group was 6.5%/year.	
Chien-Chia Wu et al. *Journal of Cancer* (2020)	Novel Oral Anticoagulant versus Warfarin in Cancer Patients with Atrial Fibrillation: An 8-Year Population-Based Cohort Study	933	Restrospective Population-Based Cohorty Study.	Ischemic stroke /systemic embolism, GI bleeding, major bleeding, intracranial hemorrhage, acute myocardial infarction , and death from any cause at 6 months and 1 year.	In cancer patients with AF, NOAC were associated with significant reduced IS/SE, major bleeding, and ICH compared to warfarin.	Medium
Deitelzweig et al. *J A C C : CARDI O ONCOLOGY* (2021)	Effectiveness and Safety of Oral Anticoagulants Among Nonvalvular Atrial Fibrillation Patients With Active Cancer	40271	Retrospective observational study	Comparison the risk of stroke/systemic embolism (stroke/SE) and major bleeding (MB) among NVAF patients with active cancer who were prescribed non–vitamin K antagonist oral anticoagulants (NOACs) or warfarin.	Compared with warfarin, apixaban was associated with a lower risk of stroke/SE (hazard ratio [HR]: 0.59; 95% confidence interval [CI]: 0.45-0.78) and MB (HR: 0.58; 95% CI: 0.50-0.68). Risks of stroke/SE and MB varied among NOAC-NOAC comparisons, while consistent treatment effects were seen for all treatment comparisons across key cancer types.	Medium

Authors, Journal of publication	Title	Sample size (N)	Study design	Variable-outcome	Main findings	Quality of evidence
Ording et al. *Cancer Medicine* (2021)	Bleeding complications in patients with gastrointestinal cancer and atrial fibrillation treated with oral anticoagulants.	2128	Nationwide cohort study comparing the bleeding risk associated with DOAC versus VKA in patients with AF and gastrointestinal (GI) cancer.	Crude bleeding rates per 100 person-years (PYs) for GI and major bleeding; comparison rates of bleeding at 1 year after initial oral anticoagulation filled prescription by treatment regimen using inverse probability of treatment weighting and Cox regression.	1-year risk of bleeding associated with DOAC was comparable with VKA users among patients with AF and GI cancer.	Medium
Potter et al. *J A C C : CARDI O ONCOLOGY* (2022).	Outcomes by Class of Anticoagulant Use for Nonvalvular Atrial Fibrillation in Patients With Active Cancer	1133	Retrospective electronic medical record review.	Rates of cerebrovascular accident (CVA), gastrointestinal bleeding (GIB), and intracranial hemorrhage (ICH) in patients treated with direct oral anticoagulant agents (DOACs) compared with warfarin for NVAF in patients with active cancer.	Comparing warfarin with DOACs, there were similar risks for CVA (subdistribution HR: 0.738; 95% CI: 0.334-1.629); ICH (subdistribution HR: 0.295; 95% CI: 0.032-2.709); GIB (subdistribution HR: 1.819; 95% CI: 0.774-4.277); and the composite of GIB, CVA, or ICH (subdistribution HR: 1.151; 95% CI: 0.645-2.054).	High

Table S2. A. Risk of bias.

Study, year of publication	Random sequence generation	Allocation concelament	Binding participants and personell	Binding of outcome	Incomplete outcome data	Selective reporting	Other bias
Fanola et all 2018,	high risk	Low risk	Low riks	High risk	Uncelar risk	Low risk	Low risk

Melloni, et all 2017	High risk	Low risk	Low risk	High risk	Uncelar risk	Low risk	Low risk
Chen et all 2019	High risk	Low risk	Low risk	High risk	Unclear risk	Low risk	Low risk

Table S2B. Newcastle Ottawa scale (NOS).

Study, year of publication	Selection	Comparability	Outcome	Quality assessment
Chan et all 2021,	***	+	+++	Fair
Deitelzweig, et all 2021	++++	+++	+++	Good
Chien-Chia Wu et all 2020	+++	+	+++	Fair
Shah et all, 2017	++++	++	+++	Good
Kim et all, 2018	++++	++	+++	Good
Sawant et all, 2019	+++	+	++	Fair
Yasui et all, 2019	+++	+	++	Fair
Ording et all, 2021	+++	+	++	Fair

Table S3. Clinical profile of patients from the included studies according to oral anticoagulant therapy (NOAC vs. Warfarin).

	VKA	NOAC
**AGE (years)**	74,7	74,7
**MALE (%)**	61%	60%
**FEMALE (%)**	39%	40%
**CHA_2DS_2-VASc SCORE**	3,82	3,88
**HAS-BLED**	2,24	2,39
**CONCOMITANT ANTIPLATELET AGENT (%)**	6,7%	8,1%

Table S4. P-Values of meta-regressions between OR (vertical column of the table) and clinical variables (horizontal column). Significant meta-regressions (P-Values <0.05) are reported in the dedicated graphs.

Metaregression p-value	Age	Female Sex	Hasbled	Chadvasc	Antiplatelets
**Outcomes**					
**Ischemic Stroke**	0,261	0,700	0,395	0,451	0,941
Minor bleeding	0,753	0,001	0,001	0,017	0,001
Major bleeding	0,164	0,030	0,693	0,407	0,198
Gastrointestinal bleeding	0,735	0,606	0,003	0,004	NA
Cerebral bleeding	0,542	0,957	0,037	0,010	0,816
